# Real-world trough concentrations and effectiveness of long-acting cabotegravir and rilpivirine: a multicenter prospective observational study in Switzerland

**DOI:** 10.1016/j.lanepe.2023.100793

**Published:** 2023-12-13

**Authors:** Paul Thoueille, Susana Alves Saldanha, Fabian Schaller, Eva Choong, Aline Munting, Matthias Cavassini, Dominique Braun, Huldrych F. Günthard, Katharina Kusejko, Bernard Surial, Hansjakob Furrer, Andri Rauch, Mathieu Rougemont, Pilar Ustero, Alexandra Calmy, Marcel Stöckle, Catia Marzolini, Caroline Di Benedetto, Enos Bernasconi, Patrick Schmid, Rein Jan Piso, Pascal Andre, François R. Girardin, Monia Guidi, Thierry Buclin, Laurent A. Decosterd, The Swiss HIV Cohort Study

**Affiliations:** a Service and Laboratory of Clinical Pharmacology, Department of Laboratory Medicine and Pathology, Lausanne University Hospital and University of Lausanne, Lausanne, Switzerland; b Service of Infectious Diseases, Department of Medicine, Lausanne University Hospital and University of Lausanne, Lausanne, Switzerland; c Department of Infectious Diseases and Hospital Epidemiology, University Hospital Zurich, Zurich, Switzerland; d Institute of Medical Virology, University of Zurich, Zurich, Switzerland; e Department of Infectious Diseases, Inselspital, Bern University Hospital, University of Bern, Bern, Switzerland; f Primary Care Medicine Division, University Hospital Geneva, Geneva, Switzerland; g Division of Infectious Diseases, Geneva University Hospitals, Faculty of Medicine, Geneva, Switzerland; h Department of Medicine, Faculty of Medicine, University of Geneva, Geneva, Switzerland; i Division of Infectious Diseases and Hospital Epidemiology, University Hospital Basel, University of Basel, Basel, Switzerland; j Department of Molecular and Clinical Pharmacology, Institute of Translational Medicine, University of Liverpool, Liverpool, UK; k Division of Infectious Diseases, Ente Ospedaliero Cantonale, Lugano, Switzerland; l University of Geneva, University of Southern Switzerland, Lugano, Switzerland; m Division of Infectious Diseases and Hospital Epidemiology, Cantonal Hospital St Gallen, St Gallen, Switzerland; n Department of Internal Medicine, Infectious Diseases and Hospital Epidemiology, Cantonal Hospital Olten, Switzerland; o Centre for Research and Innovation in Clinical Pharmaceutical Sciences, Lausanne University Hospital and University of Lausanne, Lausanne, Switzerland; p Institute of Pharmaceutical Sciences of Western Switzerland, University of Geneva, University of Lausanne, Geneva, Switzerland

**Keywords:** Long-acting cabotegravir and rilpivirine, Real-world, Drug concentration monitoring

## Abstract

**Background:**

The efficacy and tolerability of long-acting cabotegravir and rilpivirine were demonstrated in Phase III trials. However, low concentrations combined with other risk factors have been associated with an increased risk of virologic failure. This study aims to verify whether drug concentrations measured in a real-world setting are consistent with those previously reported.

**Methods:**

SHCS-879 is a nationwide observational study within the Swiss HIV Cohort Study for the monitoring of people with HIV (PWH) on long-acting cabotegravir plus rilpivirine. Samples were collected from March 2022 to March 2023.

**Findings:**

Overall, 725 samples were obtained from 186 PWH. Our data show a large inter-individual variability in cabotegravir and rilpivirine concentrations, with some individuals exhibiting repeatedly low concentrations. Rilpivirine trough concentrations were consistent with those from Phase III trials, while cabotegravir concentrations were lower. The first concentrations quartile was only slightly above the target of 664 ng/mL. Exploratory statistical analyses found 35% (p < 0·01) lower cabotegravir trough in males compared to females. Overall, 172 PWH (92%) remained suppressed and three experienced virologic failures (1·6%), of those, two had sub-optimal drug exposure. No association was found between low trough levels and detectable viral load.

**Interpretation:**

Real-world cabotegravir concentrations are substantially lower than previously reported. However, these concentrations appear sufficient to ensure sustained virological suppression in almost every PWH. These reassuring data challenge the rather conservative thresholds adopted to date, which may raise unnecessary concerns. Yet, our study reveals that some PWH have repeatedly very low drug levels, for reasons that remain to be elucidated.

**Funding:**

This work was funded by the 10.13039/501100001711Swiss National Science Foundation, grant number N^◦^ 324730_192449. This study received no support from pharmaceutical industries. This study was performed within the framework of the Swiss HIV Cohort Study, supported by the 10.13039/501100001711Swiss National Science Foundation (grant #201369), by SHCS project #879, and by the SHCS research foundation. The SHCS data were gathered by the Five Swiss University Hospitals, two Cantonal Hospitals, 15 affiliated hospitals and 36 private physicians (listed in http://www.shcs.ch/180-health-care-providers).

**Research in context**

**Evidence before this study**

Cabotegravir in association with rilpivirine is the first approved long-acting injectable regimen for the treatment of HIV infection. This new paradigm for antiretroviral delivery can pave the way to a better quality of life for people with HIV (PWH) by reducing stigma, fear of disclosure, and anxiety surrounding treatment adherence. We searched PubMed on June 21st 2023 for studies reporting cabotegravir and rilpivirine concentrations. Although long-term efficacy and tolerability were demonstrated in Phase III clinical trials, variable exposures were observed with the intramuscular administration of cabotegravir and rilpivirine. The ATLAS-2M and FLAIR pivotal studies reported significant variability in plasma concentrations of cabotegravir and rilpivirine. In particular, it was suggested that lower concentrations could potentially contribute to virologic failure and could lead to the development of resistance to the integrase inhibitor drug class. Multivariable analyses using pooled data from participants in FLAIR, ATLAS, and ATLAS-2M explored the factors predictive of virologic failure. The analysis revealed that low plasma drug concentrations in the presence of other risk factors (i.e., virus subtype, mutations associated with rilpivirine resistances and body-mass index ≥30 kg/m^2^) were associated with an increased risk of virologic failure particularly if two or more risk factors were present.

**Added value of this study**

This is the first large multicenter observational study measuring systematically plasma concentrations of injectable cabotegravir and rilpivirine during the loading and maintenance phase of treatment in PWH of the Swiss HIV Cohort Study receiving this treatment. Long-acting cabotegravir plus rilpivirine have been mainly used in the stringent framework of clinical trials, which do not account for uncertainties (i.e., delayed visits, variation in the injection technique, broader socio-demographic representation) that occur in real-world. Our findings indicate that cabotegravir plasma levels are lower than previously reported. In addition, there is a considerable variability in cabotegravir and rilpivirine exposure, with some individuals exhibiting repeatedly low concentrations. Lastly, exploratory statistical analyses revealed sex differences as males were shown to have significantly lower trough concentrations of cabotegravir compared to females.

**Implications of all the available evidence**

Although cabotegravir concentrations are significantly lower than those reported in Phase III studies, our observations support the high effectiveness of long-acting cabotegravir and rilpivirine also in a real-world setting. In fact, despite three virologic failure with no drug resistance mutations, 92% of PWH remained fully virologically suppressed. The remaining showed only occasional viral blips during the 12-month follow-up period. Yet, some individuals repeatedly had very low plasma cabotegravir and/or rilpivirine concentrations, which may be of concern and for which no clear explanation was found at the time. The relationship between repeated low levels and occurrence of detectable viral load remains unclear. Further studies are needed to understand the reason for these low concentrations and the implications for clinical care. The clinical usefulness for therapeutic drug monitoring in the context of long-acting antiretroviral therapy remains to be established.

## Introduction

Antiretroviral therapy has considerably improved over years resulting in better management of HIV infection, recently culminating in the development of the first long-acting formulation of cabotegravir and rilpivirine. After an optional 1-month oral lead-in period, a loading dose of cabotegravir and rilpivirine is injected intramuscularly. Maintenance therapy is then administered either every 2 months or every month.^1^, ^2^, ^3^ Significant variability in plasma concentrations of cabotegravir and rilpivirine has been reported in Phase III registrational studies. Preliminary observations suggested that lower concentrations of cabotegravir and rilpivirine could possibly contribute to virologic failures (VF), with the risk of developing resistance notably to the integrase inhibitor drug class.^4^ More recently, multivariable analyses revealed that rilpivirine resistance mutations at baseline, HIV-1 subtype A6/A1, body-mass index (BMI) above 30 kg/m^2^, or low rilpivirine and or cabotegravir trough concentration (C_trough_) 4 weeks after the initial loading dose were associated with an increased risk of VF.^2^^,^^3^^,^^5^^,^^6^ Specifically, there were indications that PWH with at least two risk factors may be at higher risk for VF.^2^^,^^3^

The protein-adjusted concentrations required for 90% inhibition of viral replication (PAIC_90_) with cabotegravir and rilpivirine are 166 ng/mL and 12 ng/mL, respectively, as they are characterized by extensive protein binding.^7^ However, the clinical target thresholds are higher (i.e., 4xPAIC_90_)^8^^,^^9^ based on available drug exposure-response studies. A threshold of 50 ng/mL after oral rilpivirine administration was recommended as the minimum concentration to ensure optimal therapeutic response in a population pharmacokinetic analysis.^9^^,^^10^ However, other authors have concluded that even higher plasma levels of rilpivirine should be targeted.^11^ For cabotegravir, the 664 ng/mL (4xPAIC_90_) threshold was reported to be associated with high protective efficacy in rectal and vaginal simian HIV challenge models.^8^ This target also corresponds to the 5th percentile of cabotegravir C_trough_ obtained with the first loading injection as observed in pre-exposure prophylaxis (PrEP) studies and Phase III trials.^4^^,^^12^ Of interest, Overton et al.^13^ reported in the results of the 152-week ATLAS-2M study that participants with VF had plasma C_trough_ mostly in the first quartile (Q1_Ctrough_, 25th percentile). When considering the pooled C_trough_ from Phase III trials, the Q1_Ctrough_ thresholds are 32 ng/mL and 1120 ng/mL for rilpivirine and cabotegravir, respectively.^4^^,^^5^^,^^13^

Phase III trials reported extensive pharmacokinetic variability in carefully selected study participants and found that low drug exposure, together with other risk factors, could be associated with VF. On the other hand, population pharmacokinetic analyses indicated that BMI and sex had a significant impact on cabotegravir pharmacokinetics,^14^ while no covariates were identified for rilpivirine.^15^ In particular, the absorption rate constant of cabotegravir was found to be more than 50% slower in females compared to males.^14^^,^^16^ The flip-flop kinetics inherent to the intramuscular administration of cabotegravir and rilpivirine^14^^,^^15^ implies that the rate of absorption is the limiting factor for drug elimination. Thus, a slower absorption rate implies lower peak concentrations but higher C_trough_. Therefore, females are expected to have higher cabotegravir C_trough_ than males over time. A slow absorption in women was also demonstrated by the PrEP HPTN084 study. This suggests that females may be at lower risk for subtherapeutic exposure.^17^

Altogether, these observations raise the question of whether the variability of cabotegravir and rilpivirine concentrations could be greater outside of clinical trials, where diverse clinical situations may have an impact on therapeutic responses. To address this gap, we conducted a real-world implementation study for long-acting cabotegravir and rilpivirine. The present paper aims were to provide real-world medium-term observations on long-acting treatment with cabotegravir plus rilpivirine outside the stringent framework of clinical trials. Our primary objective was to determine the proportion of real-world measurements below the previously reported thresholds for C_trough_ (i.e., PAIC_90_, 4xPAIC_90_ and Q1_Ctrough_). Our second exploratory objectives were to assess the influence of sex, BMI and albumin levels on the C_trough_ of cabotegravir and rilpivirine, and to investigate the relationship between low C_trough_ and the occurrence of detectable HIV RNA.

## Methods

### Study design and population

The SHCS, established in 1988, is a systematic longitudinal study for the follow-up of PWH in Switzerland.^18^ This organization includes all Swiss University Hospital infectious disease outpatient clinics, as well as two large canton hospitals, plus affiliated hospitals, private physicians, and laboratories.

Following the approval of long-acting cabotegravir and rilpivirine in Switzerland in March 2022, all adults (>18 years old) receiving long-acting cabotegravir and rilpivirine were invited to participated in SHCS-879, with no exclusion criteria. Participants in the SHCS give general consent at cohort inclusion for the use of their clinical data for research purposes. A small number of SHCS participants were receiving cabotegravir and rilpivirine for compassionate use prior to Swiss market authorization. A few additional samples from PWH not included in the SHCS were collected as part of their medical care, and their results were subsequently anonymized and pooled with the study data. Finally, consenting PWH participated in the detailed pharmacokinetic observations described thereafter, after approval of the corresponding study protocol by the respective ethics committee (Project-ID 2022-00619, Commission cantonale d’éthique de la recherche sur l’être humain, Lausanne, Switzerland).

### Procedures

Treatment was initiated either with an oral lead-in period or a direct intramuscular loading injection.^1^, ^2^, ^3^ In either case, a blood sample was drawn just before the loading injection. Blood samples were then scheduled to be collected every 2 months at each follow-up medical visit (i.e., at trough). Blood sampling was also repeated at unselected times in PWH whose most recent C_trough_ deviated largely from those observed in the general study population. In addition, individuals followed in Lausanne and Geneva were offered the possibility to participate in a detailed pharmacokinetic observation by providing three additional samples collected over the dosing interval at intermediate time points after drug injections (i.e., one-, two- and four-weeks post-dose).

Each blood sample was documented with the date and time of blood collection and of the last cabotegravir and rilpivirine administration including the dose, as well as demographic parameters, adverse events, and details on the injection. The choice of needle length was left to the discretion of physicians based on the Swiss prescription information. All data extracted from the report forms were entered in an electronic form developed in-house on the REDCap® platform (https://www.project-redcap.org/). Finally, relevant clinical and demographic information, such as age, sex at birth, CD4 count, viremia, and comedications, were retrieved from the SHCS database. The timing of viral load measurement was not standardized, but left to the discretion of the medical staff as part of regular clinical care. Data management, visualization and statistical analyses were performed with R (RStudio, v4.0.2, http://www.r-project.org/).

### Laboratory measurements

Blood samples were sent to the Laboratory of Clinical Pharmacology in Lausanne (Switzerland) at room temperature, shipment condition wherein cabotegravir and rilpivirine have been experimentally demonstrated to be stable. Blood samples were centrifuged at +4 °C and the collected plasma stored at −80 °C prior to drug analyses, performed within one week. Drug concentrations in plasma were measured by multiplex high performance liquid chromatography coupled to tandem mass spectrometry using a validated multiplex method.^19^ The laboratory participates in International External Quality Control Proficiency Programs for cabotegravir and rilpivirine (Asqualab, Paris, France; KKGT, The Hague, The Netherlands). The analyses were performed once a week and the drug concentration results were sent to the treating physician within the same week. Due to the observational nature of the study, the decision to discontinue treatment based on the results of the drug measurements was left to the care provider. Finally, plasma albumin levels were measured (to reflect drug binding and distribution) using a colorimetric analytical method developed by Unilabs (https://unilabs.ch/fr).

### Outcomes

The primary study outcome was the proportion of cabotegravir and rilpivirine C_trough_ above the following reported thresholds: PAIC_90_ (166 ng/mL for cabotegravir, 12 ng/mL for rilpivirine), 4xPAIC_90_ (664 ng/mL for cabotegravir, 50 ng/mL for rilpivirine), and Q1_C___trough__ (1120 ng/mL for cabotegravir, 32 ng/mL for rilpivirine). Comprehensive population pharmacokinetic-pharmacodynamic analyses of the entire study population are underway but are out of the scope of this manuscript.

Secondary exploratory objectives included the identification of predictive factors (i.e., sex, BMI and albumin levels) of cabotegravir and rilpivirine concentrations and the association of “on-target” plasma levels with treatment efficacy and tolerability. The project also measured the pharmacokinetic tail for PWH who interrupted intramuscular injections, and explored factors associated with viral failure (defined as HIV viral load above 200 copies/mL).

### Exploratory statistical analyses

Proportions above/below the PAIC_90_, 4xPAIC_90_ and Q1_Ctrough_ thresholds were calculated both among the total number of samples collected, and at the individual level (with and without stratification by sex). Based on the previously published analyses^14^, ^15^, ^16^ and the data collected, a multivariable linear mixed-effect regression was performed to assess whether sex, BMI, and albumin plasma levels significantly influenced drug concentrations, taking into account treatment week. BMI and albumin plasma levels were centered on their median. We tested models with time and individuals as both random intercepts, and random intercepts and slopes. The intraclass correlation coefficient was estimated using the final mixed-effect model. We also explored the association with each covariate individually. In accordance with the median duration of follow-up, the analysis was conducted on the C_trough_ values measured over the first 32 weeks of treatment. Inter- and intra-individual variabilities were derived from the standard deviations of random effects and residual errors. We did not perform formal sample size calculations for this exploratory study aiming at describing the real-world pharmacokinetics of long-acting antiretrovirals. Finally, the association between C_trough_ and HIV RNA was assessed by logistic regression, taking both usual detection thresholds of 20 and 50 copies/mL. Because the frequency of viral load measurement was not standardized, the analysis was performed with the most recent viral load value recorded before each drug concentration value. Intra-patient correlation was not taken into account because the vast majority of PWH remained undetectable. Furthermore, only a few PWH had more than one viral load above the usual threshold of 50 copies/mL.

### Role of the funding source

The public funding source of the study had no role in the design of the study, data collection, data analysis, data interpretation, manuscript writing, or decision to publish.

## Results

### Study population

One year after the study initiation (March 2022–March 2023), 186 PWH were included in SHCS-879. Table 1 summarizes their characteristics.^20^, ^21^, ^22^ Participants were mostly male (82%), white (57%) with a median age of 45 (range: 20–79). On the other hand, of the 33 females included so far, 52% were black with a median age of 44 years (20–63). Median BMI was 25.5 kg/m^2^, and 28 PWH (15%) were obese (BMI ≥30 kg/m^2^), without significant sex differences in the distribution.

**Table 1:** Population characteristics of PWH.

Population characteristics *Last measured value*	Median (range) or n (%)		
	*Overall (n = 186)*	*Males (n = 153)*	*Females (n = 33)*
**Demographics and clinics**			
Age, years	45 (20–79)	46 (26–79)	44 (20–63)
Ethnicity			
White	106 (57%)	98 (64%)	8 (24%)
Black	32 (17%)	15 (10%)	17 (52%)
Hispanic American	14 (8%)	13 (9%)	1 (3%)
Asian	7 (4%)	6 (4%)	1 (3%)
Other	1 (<1%)	–	1 (3%)
Missing	26 (14%)	21 (11%)	5 (15%)
Body weight, kg	78 (52–126)	80 (52–126)	70 (57–102)
Height, cm	176 (151–198)	178 (151–198)	165 (151–180)
BMI, kg/m^2^			
Range	25.5 (17.6–37.5)	25.2 (17.6–37.5)	26.5 (19.0–34.1)
PWH with BMI ≥ 30	28 (15%)	23 (15%)	5 (15%)
eGFR^a^, mL/min/1.73m^2^^20^			
G1: ≥90	118 (63%)	97 (63%)	21 (64%)
G2: 60–89	61 (33%)	50 (33%)	11 (33%)
G3a: 45–59	3 (2%)	3 (2%)	–
Liver cirrhosis^a^, Child-Pugh score^21^			
No	180 (97%)	150 (98%)	30 (91%)
Class A	2 (1%)	–	2 (6%)
Heart failure^a^, NYHA scale^22^			
No	181 (97%)	149 (97%)	32 (97%)
Class I	1 (<1%)	1 (<1%)	–
Albumin plasma level (g/L)	46.0 (38.4–57.8)	46.2 (38.4–57.8)	44.3 (38.8–52.6)
**Disease characteristics**			
Years since HIV diagnosis	10.4 (0.7–37.2)	10.1 (0.7–36.8)	12.0 (3.0–37.2)
Missing	24 (13%)	19 (12%)	5 (15%)
CD4 cell count^a^, cells/μL			
≥500	155 (83%)	129 (84%)	26 (79%)
350 to <500	19 (10%)	13 (8%)	6 (18%)
<350	9 (5%)	8 (5%)	1 (3%)
Plasma HIV RNA^a^, copies/mL			
<50	177 (95%)	146 (95%)	31 (94%)
≥50 and <200	4 (2%)	3 (2%)	1 (3%)
≥200	3 (1.6%)	2 (1.3%)	1 (3%)
**Antiretroviral therapy and comedications**			
PWH who received oral lead-in	172 (92%)	143 (93%)	29 (88%)
Duration of follow-up, weeks	24 (2–188)	25 (2–188)	16 (2–98)
Previous antiretroviral therapy			
INSTI	117 (63%)	103 (67%)	14 (42%)
NRTI	149 (80%)	123 (80%)	26 (78%)
NNRTI	27 (15%)	18 (12%)	9 (27%)
PI	8 (4%)	4 (3%)	4 (12%)
Missing	35 (19%)	28 (18%)	7 (21%)

BMI: body-mass index; eGFR: estimated Glomerular Filtration Rate, calculated according to the CKD-EPI equations reported by Levey et al.^20^; NYHA: New York Heart Association scale^22^; INSTI: integrase strand transfer inhibitor; NRTI: nucleoside reverse transcriptase inhibitor; NNRTI: non-nucleoside reverse transcriptase inhibitor; PI: protease inhibitor.

Note: Histograms of continuous variables are reported in Figure S1.

a ≤2% missing information.

Overall, 172 PWH (92%) remained fully virologically suppressed (HIV viral load <50 copies/mL), while 11 PWH (6%) had viral blips (isolated detectable HIV viral load <200 copies/mL) and three VF occurred (1·6%). As HIV subtype was known only for a minority of PWH, we did neither report it nor use it in any analysis.

From 186 PWH, 725 samples were collected, of which 569 were obtained during the injection phase. Table S1 summarizes the information on follow-up and sample collection. Overall, 172 PWH (92%) received an oral lead-in. It should be noted that two PWH received long-acting cabotegravir and rilpivirine every 4 weeks for compassionate use prior to Swiss market authorization, as this was the only recommended regimen at that time. These PWH were switched to the 2-month regimen a few months after the start of the study. The median duration of follow-up for males and females was 25 weeks (2–188) and 16 weeks (2–98), respectively, and 91% of injections (1056 out of 1158 injections) were performed within the recommended window of ± 7 days.^1^, ^2^, ^3^ Most levels were collected at C_trough_ (470 samples, 79%), and 27 PWH (15%) participated in the detailed pharmacokinetic investigations. Details on adverse events are reported in Table S2.

### Observed concentrations

**Fig. 1:**
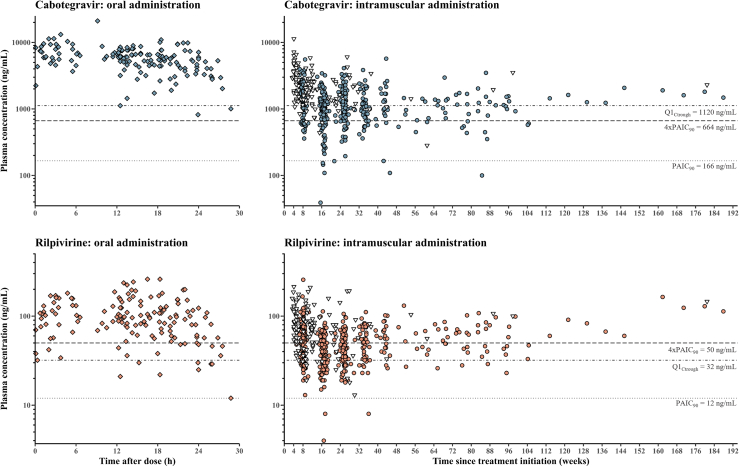
Observed plasma concentrations of cabotegravir and rilpivirine. Closed circles represent trough levels, while open triangles represent levels measured at intermediary times during the administration interval. The horizontal lines correspond to the PAIC_90_, 4xPAIC_90_ and Q1_Ctrough_ thresholds reported.^4^^,^^7^, ^8^, ^9^^,^^12^^,^^13^

**Fig. 2:**
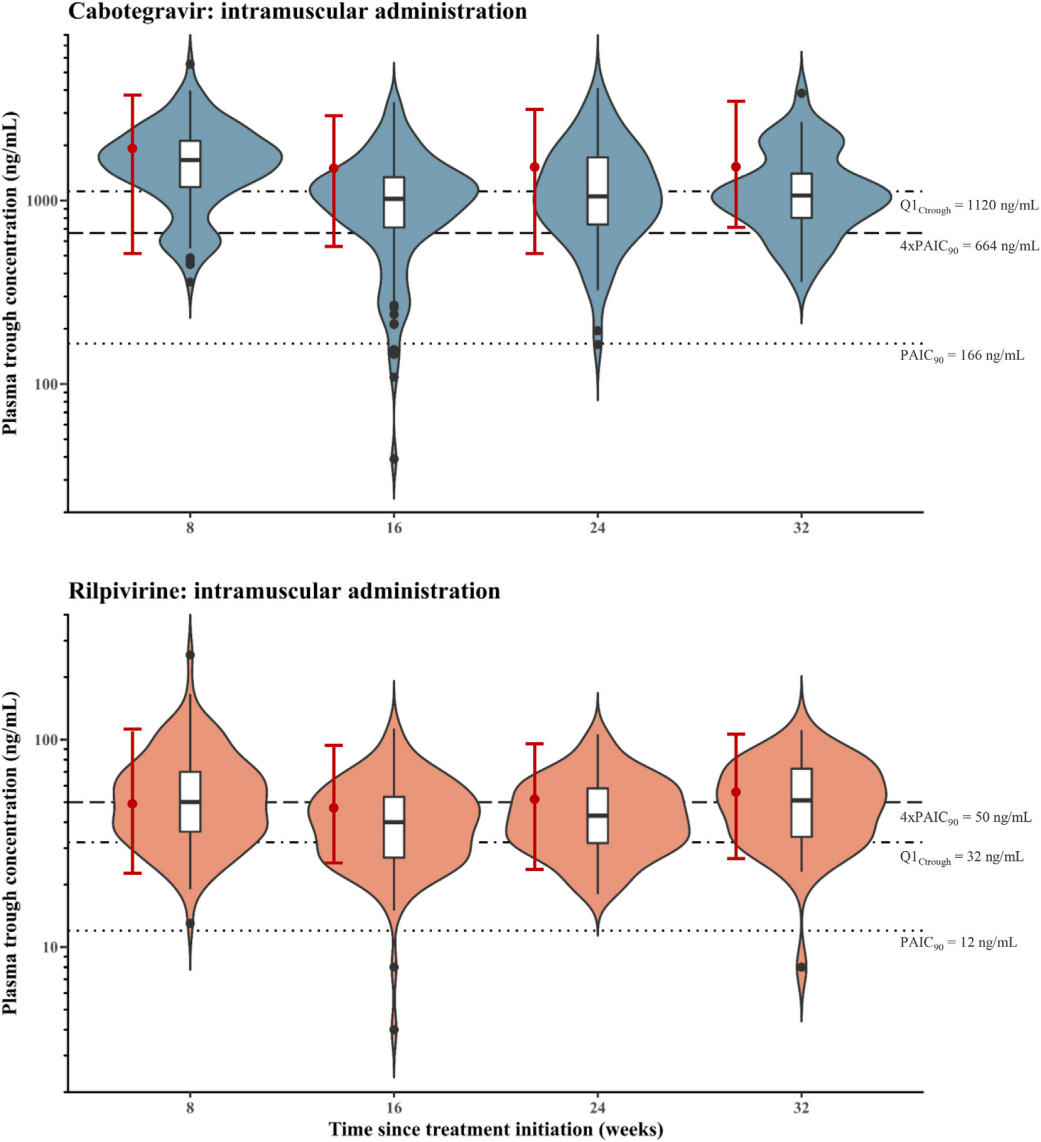
Comparison of trough plasma concentrations of cabotegravir and rilpivirine over 32 weeks. The violin plots represent the distribution of concentration data. The boxplots depict the median and interquartile range. Whiskers extend from the lowest to the highest values comprised within 1.5 times the interquartile range. Outliers are represented by black dots. Concentration ranges from the ATLAS-2M trial are shown in red bars as medians (circles), 5% and 95% percentiles.^23^ The horizontal lines correspond to the PAIC_90_, 4xPAIC_90_ and Q1_Ctrough_ thresholds reported.^4^^,^^7^, ^8^, ^9^^,^^12^^,^^13^

Fig. 1 depicts all plasma concentrations and Fig. 2 shows the C_trough_ after intramuscular injections from week 8 (i.e., 4 weeks after the first injection) to week 32, compared to the ranges reported in ATLAS-2M.^23^ Table 2 presents the percentage of observed concentrations below the three thresholds reported in literature.^4^^,^^7^, ^8^, ^9^^,^^12^^,^^13^ More than 50% of rilpivirine C_trough_ were below the target of 50 ng/mL during the 32 weeks observation period.^9^^,^^10^ However, our rilpivirine concentrations were consistent with those reported in Phase III trials (i.e., first quartile of observed C_trough_ is around 32 ng/mL).^4^^,^^13^ Of note, about 7% of rilpivirine levels were lower than twice the PAIC_90_ of 12 ng/mL,^7^ while two PWH (1%) had rilpivirine concentrations below the PAIC_90_.

**Table 2:** Comparison of observed concentrations of cabotegravir and rilpivirine with reported thresholds.

Observed concentrations	Number (%)		
	*Overall* (*569 samples*, 186 PWH)	*Males* (*486 samples*, 153 PWH)	*Females* (*83 samples*, 33 PWH)
**Cabotegravir q8w**			
**Plasma concentrations**			
<1120 ng/mL (Q1_Ctrough_)^a^	210 (37%)	195 (40%)	15 (18%)
<664 ng/mL (4xPAIC_90_)^b^	83 (15%)	75 (15%)	8 (10%)
≤166 ng/mL (PAIC_90_)	8 (1%)	8 (2%)	–
**PWH with ≥1 measurement**			
<1120 ng/mL (Q1_Ctrough_)^a^	106 (57%)	94 (61%)	12 (36%)
<664 ng/mL (4xPAIC_90_)^b^	56 (30%)	48 (31%)	8 (24%)
≤166 ng/mL (PAIC_90_)	8 (4%)	8 (5%)	–
**PWH with ≥2 measurements**			
<1120 ng/mL (Q1_Ctrough_)^a^	57 (31%)	55 (36%)	2 (6%)
<664 ng/mL (4xPAIC_90_)^b^	18 (10%)	18 (12%)	–
≤166 ng/mL (PAIC_90_)	–	–	–
**Rilpivirine q8w**			
**Plasma concentrations**			
<50 ng/mL (4xPAIC_90_)^c^	273 (48%)	231 (48%)	42 (51%)
<32 ng/mL (Q1_Ctrough_)^a^	104 (18%)	81 (17%)	23 (28%)
≤12 ng/mL (PAIC_90_)	3 (<1%)	–	3 (4%)
**PWH with ≥1 measurement**			
<50 ng/mL (4xPAIC_90_)^c^	119 (64%)	99 (65%)	20 (61%)
<32 ng/mL (Q1_Ctrough_)^a^	60 (32%)	47 (31%)	13 (39%)
≤12 ng/mL (PAIC_90_)	2 (1%)	–	2 (6%)
**PWH with ≥2 measurements**			
<50 ng/mL (4xPAIC_90_)^c^	76 (41%)	66 (43%)	10 (30%)
<32 ng/mL (Q1_Ctrough_)^a^	25 (13%)	22 (14%)	3 (9%)
≤12 ng/mL (PAIC_90_)	1 (<1%)	–	1 (3%)

PWH: people with HIV; BMI: body-mass index; q8w: every 8 weeks.

a Correspond to the limit of the first quartile (25th percentile) when considering all rilpivirine and cabotegravir concentrations observed in phase III FLAIR^4^ and ATLAS.^13^

b Corresponds to 4xPAIC_90_,^8^ and corresponds also to the 5th percentile of cabotegravir long-acting initial C_trough_ observed in PrEP prevention studies.^12^

c Minimum concentration to ensure optimal therapeutic response.^9^^,^^10^

Cabotegravir C_trough_ were markedly lower than the levels previously reported in Phase III trials (see Fig. 2). From week 16 to week 32, more than 50% of cabotegravir concentrations were below the Q1_Ctrough_ threshold of 1120 ng/mL.^4^^,^^13^ The first quartile of cabotegravir C_trough_ in our real-world study was only slightly above the recommended 4xPAIC_90_ (664 ng/mL) during the 32 weeks observation period.^8^ Cabotegravir levels below the PAIC_90_ of 166 ng/mL^7^ were observed in 8 males (4%), two of whom were obese.

Overall, no interacting comedications, such as CYP3A4 or UGT1A1 inducers, were reported. Lastly, Table S3 compares the concentrations observed in obese PWH with the thresholds reported.

Table 3 summarizes the characteristics of the three PWH who experienced virologic failure and reports the timeline of injections. One virologic failure occurred despite having drug concentrations in the expected range (Table 3, ID1). Conversely, the two other virologic failures were observed in PWH with low to very low drug concentrations at multiple time points (Table 3, ID2 and ID3), of those one was obese. None of these individuals developed mutations associated with drug resistance.

**Table 3:** Characteristics of the PWH who experienced virologic failure with the timeline of injections.

ID	Sex	Age (years)	BMI (kg/m^2^)	Timing of the blood sampling	Cabotegravir concentration (ng/mL)	Rilpivirine concentration (ng/mL)	HIV RNA (copies/mL)
1	Male	49	24	4 weeks after injection n°1, at C_trough_	2445	90	<50
				8 weeks after injection n°2, at C_trough_	1151	52	17,000
				4 weeks after injection n°3	2751	114	<50
				6 weeks after injection n°3	1128	77	<50
				*Switch to bictegravir/emtricitabine/tenofovir alafenamide 6 weeks after injection n°3*			<50
				10 weeks after injection n°3	120	65	<50
2	Male	33	24	2 weeks after injection n°1	1916	77	<50
				4 weeks after injection n°1, at C_trough_	1441	53	<50
				8 weeks after injection n°2, at C_trough_	712	25	<50
				8 weeks after injection n°3, at C_trough_	823	33	<50
				8 weeks after injection n°4, at C_trough_	1049	45	<50
				8 weeks after injection n°5, at C_trough_	109	36	<50
				8 weeks after injection n°6, at C_trough_	696	38	<50
				8 weeks after injection n°7, at C_trough_	704	55	549
3	Female	48	31	8 weeks after injection n°3, at C_trough_	239	4	62,000
				*Switch to emtricitabine/tenofovir alafenamide/raltegravir*			139
				*Re-initiation of cabotegravir and rilpivirine* 4 months *later*			<50
				4 weeks after injection n°1, at C_trough_	272	13	70
				3 weeks after injection n°2	402	19	<50
				8 weeks after injection n°2	–	–	<50
				*Switch to doravirine/lamivudine/tenofovir disoproxil fumarate 7 weeks after injection n°4 due to persistent low-level viremia*			61

Alternately, long-acting therapy was preemptively discontinued in three PWH with repeatedly low concentrations due to the concerns about the risk of developing resistance. These PWH did not experience virologic failure. Specifically, one woman had rilpivirine levels repeatedly below the PAIC_90_ over three administration intervals without detectable HIV RNA. In the second case, a man had persistent low cabotegravir C_trough_ around 400 ng/mL, coincident with an increase in HIV RNA (from <20 to 40, then 47 copies/mL). This individual also had continuously rilpivirine C_trough_ less than two-times the PAIC_90_. Finally, another man had consecutive cabotegravir C_trough_ barely above the PAIC_90_ leading to discontinuation of treatment, although no HIV RNA was detectable.

### Exploratory statistical analyses

The cabotegravir C_trough_ levels were 35% [95% confidence interval: −47 to −20] lower in males compared to females, adjusting for BMI and albumin plasma levels, confirming observations from clinical trials (Table S4).^14^^,^^16^^,^^17^ However, the cabotegravir C_trough_ levels did not vary according to BMI, neither differed significantly in obese PWH. Albumin plasma levels had no impact on cabotegravir C_trough_. None of these covariates affected rilpivirine C_trough_, consistent with previous reports.^15^ Observed concentrations revealed important inter- and intra-individual variability for both cabotegravir (31% and 52%, respectively) and rilpivirine (35% and 29%, respectively). In addition, the coefficient of intraclass correlation was estimated to be 0·26 and 0·59 for cabotegravir and rilpivirine, respectively. Finally, no definite association between C_trough_ of either cabotegravir or rilpivirine and viral load could be detected (Table S5).

## Discussion

Our one-year large observational study on injectable cabotegravir and rilpivirine shows significant pharmacokinetic variability of the same order of magnitude as previously reported in registrational studies.^4^^,^^13^ However, while rilpivirine levels were comparable with those from Phase III clinical trials, cabotegravir C_trough_ were substantially lower. It is not yet clear whether low plasma levels, associated with known or unknown risk factors, could compromise therapeutic success.^6^ However, we have observed PWH with recurrent low levels of cabotegravir and/or rilpivirine which raised concerns about the efficacy of the treatment. These individuals had no risk factors at baseline but became potentially at risk to develop virologic failure due to the significantly insufficient drug concentrations measured on several occasions. As a precautionary measure, three PWH had their treatment interrupted by medical decision.

On another note, our rate of 1·6% virological failure was in line with the rate reported by Orkin et al. (i.e., 1·4%).^6^ Among the PWH who experienced virologic failure, one individual had satisfactory drug concentrations without any risk factors (i.e., ID1). It remains unclear why the viral load increased to 17,000 copies/mL and spontaneously declined to undetectable levels within 4 weeks prior to the switch to oral antiretroviral therapy. Yet, in this patient, the sample collected after long-acting treatment discontinuation showed a surprising 10-fold decrease in cabotegravir concentrations in only four weeks. The other two VFs were observed in PWH with low to very low drug concentrations at multiple time points. One was notably observed in an obese woman (i.e., ID3). The initial low drug levels of cabotegravir and rilpivirine were initially presumed to result from the use of the standard needle (23-gauge, 0.65 mm, 38 mm) in this obese woman, thus inadvertently delivering the drugs nanoparticles into the adipose tissue and compromising drug release. A longer needle was therefore used when long-acting cabotegravir-rilpivirine therapy was re-initiated, yet without showing any significant effect on C_trough_ of cabotegravir and rilpivirine as the values were still repeatedly suboptimal. Thus, using a longer needle length may not always be sufficient, highlighting the need for more research to identify factors leading to recurrent low concentrations of cabotegravir and rilpivirine in some individuals. This individual was able to maintain virologic suppression upon re-initiation of long-acting injectable treatment after the second injection. However, treatment was eventually discontinued due to persistent low-level viremia. Unfortunately, blood samples for drug level measurements were not obtained after the second injection.

Although long-acting cabotegravir plus rilpivirine allows overcoming adherence issues, these drugs are characterized by a large inter- and intra-individual variability in exposure. Given the flip-flop kinetics, resorption (drug release from nanoparticles formulation) and absorption (local drug dissemination) appear to be the primary drivers of systemic drug exposure, and presumably also the main source of variability. Detailed pharmacokinetic sampling in some PWH revealed variable pharmacokinetic profiles, with some showing low concentrations even one- or two-weeks post injection. Whether such erratic pharmacokinetic profiles are due to either near-zero resorption at the injection site or, conversely, to extremely rapid absorption and, in turn, elimination, is the subject of ongoing investigations. Exploratory analyses suggested a non-significant association of BMI with cabotegravir C_trough_. Conversely, sex was found to be strongly associated with cabotegravir C_trough_ concentrations, as males exhibited significantly lower levels than females after the initial injections. Because BMI does not account for fat distribution or muscle anatomy,^24^ sex differences in cabotegravir resorption and/or absorption seem a more likely hypothesis to explain part of the variability in cabotegravir exposure between subjects.

Since cabotegravir and rilpivirine are highly bound to plasma proteins, mainly albumin,^1^, ^2^, ^3^ we investigated whether decreased plasma protein binding could lead to an increase in free plasma concentrations, resulting in an augmented volume of distribution and thereby a decrease in total plasma concentrations. However, the albumin levels measured in the study samples failed to demonstrate an association with total drug plasma levels. Other hypotheses are also considered. Intense physical activity in the hours or days following injections could possibly accelerate drug resorption and/or absorption. This would result in higher peak levels and lower C_trough_. Massages and topical creams can also enhance drug absorption. Finally, it is possible that some drug transporters diversely expressed in tissues relevant for intramuscular administration (skeletal muscles and subcutaneous fat^25^) as well as other genetic variants reported to modulate body composition, fat mass^26^ and muscular phenotypes^27^ could affect drug absorption in some PWH.

There is still no guidance for the interpretation of cabotegravir and rilpivirine therapeutic drug monitoring, yet various thresholds have been reported.^4^^,^^5^^,^^8^^,^^9^^,^^13^ Despite the fact that cabotegravir concentrations measured in our real-world study were lower than those reported in Phase III studies, our observations provide reassurance about the implementation of long-acting cabotegravir and rilpivirine in a real-world setting, showing that these concentrations are still sufficient to insure a sustained virological control in most PWH. Such reassuring observations challenge the rather conservative drug levels thresholds adopted to date, which may raise unnecessary alarmist concerns amongst healthcare providers. Concentrations of cabotegravir and rilpivirine similar to our observations were reported in another real-world study in 58 PWH,^28^ confirming the significant difference in pharmacokinetics between clinical trials participants and real-world PWH. It is worth mentioning that FDA initially raised some concerns about the treatment's manufacturing process (although not related to safety) before approving cabotegravir and rilpivirine. It may be possible that the formulation used (as well as split injections)^14^ prior to market authorization contributed to overall higher plasma drug levels, which may explain the observed differences in drug exposure between real-world studies and Phase III trials.

The robustness and the generalizability of these exploratory analyses might be limited given that the patients follow-up was not standardized. Clinical care was carried out in accordance with the official Swiss drug monograph, and some aspects were therefore left to the discretion of physicians (e.g., viral load monitoring, needle length, timing of blood sampling, decision to discontinue treatment). In fact, such procedures were not described in the protocol of our study, which was essentially observational and designed to collect drug concentrations, without influencing clinical care. The cautionary decision of physicians to change the treatment in some patients with repeatedly low drug concentration could have possibly influenced the correlation between low pharmacokinetic exposure and virological failure. In addition, data were collected from a moderately diverse population, thus possibly preventing the identification of further factors influencing drug disposition. Future prospective studies including ethnically diverse populations, subgroups or representing special clinical situations are warranted before our findings can be generalized.

Further clinical investigation is warranted to understand the reasons why some PWH repeatedly exhibit very low drug concentrations, and whether therapeutic drug monitoring might be useful to identify precociously those individuals at risk of not fully benefiting of long-acting therapies. In this regard, we are planning a nested case–control study to comprehensively investigate PWH having recurrent low concentrations and to identify potential factors that could explain altered absorption or clearance of long-acting antiretrovirals. Our findings also emphasize the importance of conducting case–control studies to identify further risk factors for virologic failure beyond those already reported.

## Contributors

P.T., P.A., M.G., T.B. and L.A.D. are principal investigators of the study and were responsible of the design of the study, and data interpretation. P.T., S.A.S., F.S., E.C., and L.A.D. were responsible for drug levels measurements. L.A.D. was responsible for project administration, acquisition of funding, and resources. P.T. was responsible of data collection and management, data visualization, and drafted the manuscript. P.T., M.G., and T.B. did the statistical analyses. K.K. was responsible for data management of the SHCS and provided patient-level demographic data on the study participants. A.M., M.C., D.B., H.F.G., B.S. H.F., A.R., M.R. P.U., A.C., M.S., M.B., C.M., C.B., E.B., P.S., R.J.P., and F.R.G. were responsible for the medical investigations. All authors critically reviewed and approved the final manuscript. All authors take responsibility for the integrity of the data and the accuracy of the data analysis. All authors had full access to all the data in the study and had final responsibility for the decision to submit for publication.

## Data sharing statement

A request for datasharing can be sent to the Scientific Board of the Swiss HIV Cohort Study (https://www.shcs.ch/). A detailed explanation of the purpose for the request as well as a study protocol, if applicable, should be presented. The final decision about data release will be taken by the Scientific Board of the SHCS.

## Declaration of interests

M.C. reports grants and payment for expert testimony from Gilead, MSD and ViiV, and support for attending meetings from Gilead, paid to his institution outside of the submitted work. D.B. has received honoraria for advisory board from the companies Gilead, MSD, and ViiV, and support for attending meetings from ViiV and Gilead. H.F.G. has received unrestricted research grants from Gilead Sciences; fees for data and safety monitoring board membership from Merck; consulting/advisory board membership fees from Gilead Sciences, Johnson and Johnson, Janssen, Novartis, and ViiV Healthcare; and grants from the Swiss National Science Foundation, the Yvonne Jacob Foundation, from National Institutes of Health and unrestricted research grants from Gilead Sciences. B.S. reports support for travel grants and advisory boards from Gilead Sciences and ViiV, paid to his institution outside of the submitted work. The institution of H.F. received educational grants from ViiV, MSD, AbbVie, Gilead, and Sandoz paid to the institution. M.S. reports advisory board paid to his institution by Gilead, MSD, ViiV, Moderna and Pfizer. The institution of A.R. received grants from Gilead; support for attending meetings from Gilead and Pfizer; and advisory boards from MSD and Moderna. C.M. has received speaker honoraria from ViiV, MSD, and Gilead unrelated to this work. C.D.B. received travel grant for congress participation from Gilead. The institution of E.B. received grants from the Swiss National Science Foundation; grants from MSD; support for attending meetings from Gilead, MSD, ViiV and Pfizer; and advisory boards from Gilead, MSD, ViiV, Pfizer, Moderna, AstraZeneca, Abbvie and Lilly. The institution of P.S. received honoraria for advisory board and support for attending meetings from ViiV and Gilead. The other authors declare no conflict of interest.
