# Supplementary material for: Real-world trough concentrations and effectiveness of long-acting cabotegravir and rilpivirine: a multicenter prospective observational study in Switzerland
Source: Lancet Reg Health Eur. 2023 Dec 13;36:100793. doi: 10.1016/j.lanepe.2023.100793 (PMC10757247; doi:10.1016/j.lanepe.2023.100793)
Supplement: Supplementary Figure and Tables [file mmc1.docx]

**Supplementary Materials**

[Figure S1: Histograms of continuous variables. 2](#_Toc143759436)

[Table S1: Summary of the follow-up and sample collection. 3](#_Toc143759437)

[Table S2: Summary of adverse events. 4](#_Toc143759438)

[Table S3: Comparison of observed concentrations of cabotegravir and rilpivirine in obese PWH (BMI ≥ 30 kg/m^2^) with reported thresholds. 5](#_Toc143759439)

[Table S4: Results of the mixed-effect regression on trough concentrations of cabotegravir and rilpivirine. 6](#_Toc143759440)

[Table S5: Logistic regression between detectable HIV RNA and drug concentrations at trough after intramuscular administration. 7](#_Toc143759441)


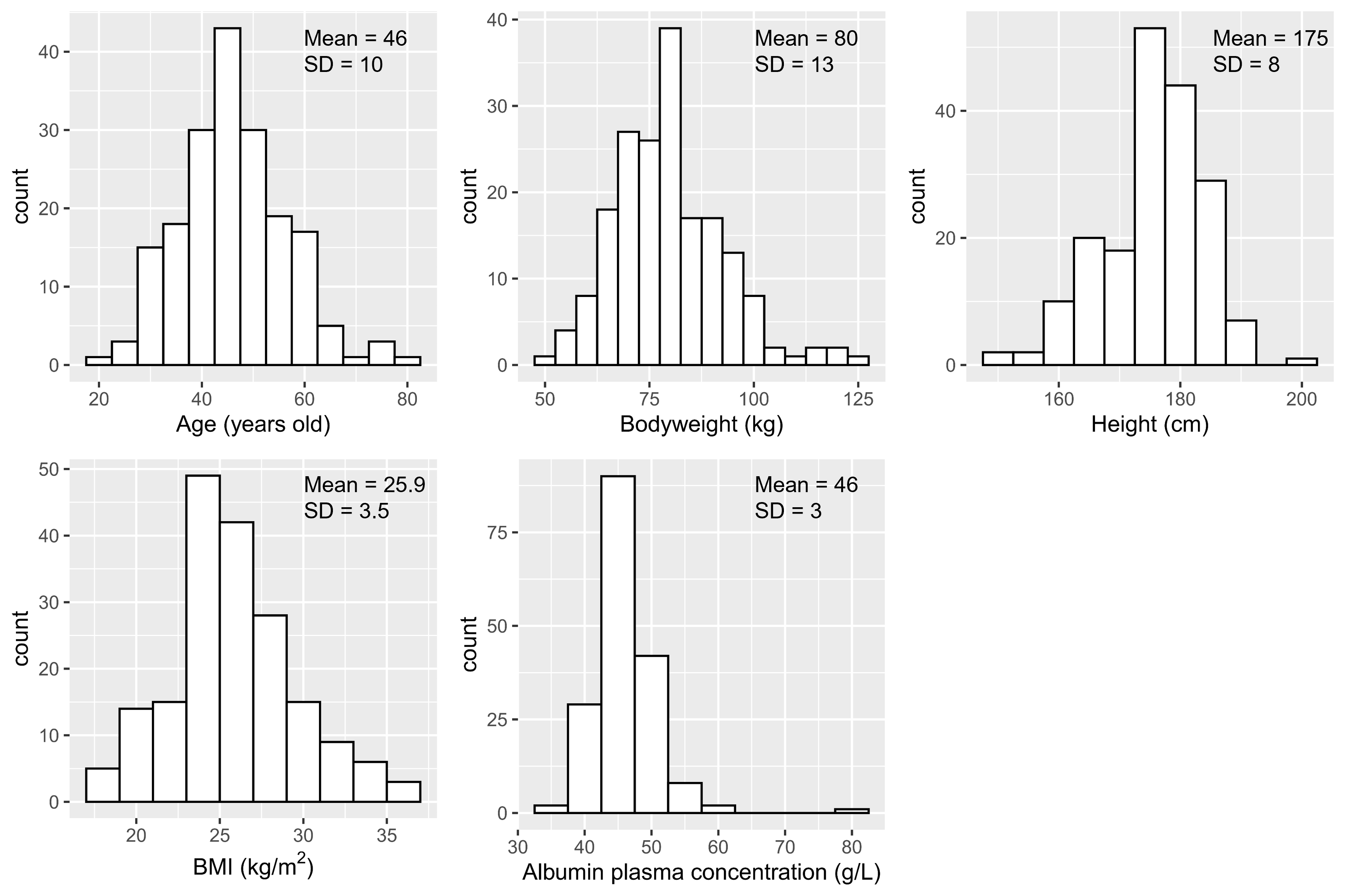


# Figure S1: Histograms of continuous variables.

# Table S1: Summary of the follow-up and sample collection.

| Follow-up | Number (% or range) | | |
| --- | --- | --- | --- |
|  | *Overall* | *Male* | *Female* |
| PWH currently receiving  Oral cabotegravir and rilpivirine  Cabotegravir and rilpivirine q8w | 18 (10%)  168 (90%) | 12 (8%)  141 (92%) | 6 (18%)  27 (82%) |
| Samples collected  During oral lead-in period  During q4w regimen *  During q8w regimen | 146 (20%)  10 (1%)  569 (79%) | 125 (20%)  10 (2%)  486 (78%) | 21 (20%)  -  83 (80%) |
| Treatment with cabotegravir and rilpivirine  Median duration of follow-up, weeks  PWH who followed oral lead-in period **  Injections within the window of +/- 7 days ^1-3^ | 24 (2 – 188)  172 (92%)  1056 (91%) | 25 (2 – 188)  143 (93%)  904 (91%) | 16 (2 – 98)  29 (88%)  152 (92%) |
| Sampling schedule (during loading dose)  ≤ 3 weeks after injections  At trough | 45 (8%)  122 (21%) | 41 (8%)  101 (21%) | 4 (5%)  21 (25%) |
| Sampling schedule (during maintenance treatment)  ≤ 2 weeks after injections  2 to 6 weeks after injections  At trough *** | 16 (3%)  58 (10%)  338 (58%) | 13 (3%)  51 (10%)  290 (58%) | 3 (4%)  7 (8%)  48 (58%) |

PWH: people with HIV; BMI: body-mass index. q4w: every 4 weeks; q8w: every 8 weeks.

* 2 PWH received long-acting cabotegravir and rilpivirine every 4 weeks for compassionate use prior to Swiss market authorization, as this was the only recommended regimen at that time. These PWH were switched to the q8w regimen few months after the start of the study.

** 4 PWH with missing information.

*** 10 plasma levels were collected at trough during the q4w regimen (2 PWH).

**Adverse events**

Table S2 summarizes the main adverse events. Overall, 75 PWH (40%) did not report any adverse events. Injection-site reactions were the most common adverse events, affecting 85 PWH (45%), but usually resolved within days. Nevertheless, two individuals had to discontinue long-acting treatment due to recurrent severe local reactions. Overall, adverse events were predominant especially during the first weeks of treatment, as previously reported.^13^

# Table S2: Summary of adverse events.

| Categories | Total reported (%)  *(n=725)* | PWH (%) | | |
| --- | --- | --- | --- | --- |
|  |  | Overall *(n=186)* | Male  *(n=153)* | Female *(n=33)* |
| No adverse events, n (%) | 480 (66%) | 75 (40%) | 62 (41%) | 13 (39%) |
| Any adverse event, n (%)  Injection site reaction ^a^  Pyrexia ^b^  Fatigue ^c^  Headache  Musculoskeletal pain ^d^  Gastro-intestinal disorders ^e^  Sleep disorders ^f^  Rash ^g^ | 245 (34%)  156 (22%)  25 (3%)  28 (4%)  9 (1%)  74 (10%)  5 (<1%)  12 (1%)  2 (<1%) | 111 (60%)  85 (46%)  16 (9%)  19 (10%)  5 (3%)  48 (26%)  5 (3%)  9 (5%)  1 (<1%) | 91 (59%)  69 (45%)  14 (9%)  16 (10%)  4 (3%)  40 (26%)  4 (3%)  8 (5%)  1 (<1%) | 20 (61%)  16 (48%)  2 (6%)  3 (9%)  1 (3%)  8 (24%)  1 (3%)  1 (3%)  - |

^a^ Includes pain/discomfort, nodules, induration, swelling, erythema, pruritis, bruising, discolouration, warmth, heamatoma.

^b^ Includes pyrexia, feeling hot, chills, influenza-like illness, body temperature increased.

^c^ Includes fatigue, malaise, asthenia.

^d^ Includes musculoskeletal pain, musculoskeletal discomfort, back pain, myalgia, pain in extremity.

^e^ Includes nausea, dizziness, diarrhea.

^f^ Includes insomnia, poor quality sleep, somnolence.

^g^ Includes erythema, pruritis generalized, purpura, rash, rash-erythematous, generalized, macular.

# Table S3: Comparison of observed concentrations of cabotegravir and rilpivirine in obese PWH (BMI ≥ 30 kg/m^2^) with reported thresholds.

| Observed concentrations  in obese PWH (BMI ≥ 30 kg/m^2^) | Number (%) | | |
| --- | --- | --- | --- |
|  | Overall  *(77 samples,*  *28 PWH)* | Males  *(61 samples,*  *23 PWH)* | Females  *(16 samples,*  *5 PWH)* |
| Cabotegravir q8w | | | |
| Plasma concentrations  < 1120 ng/mL (Q1_Ctrough_) ^a^  < 664 ng/mL (4xPAIC_90_) ^b^  ≤ 166 ng/mL (PAIC_90_) | 21 (27%)  9 (12%)  2 (3%) | 19 (31%)  7 (12%)  2 (3%) | 2 (13%)  2 (13%)  - |
| PWH with ≥ 1 measurement  < 1120 ng/mL (Q1_Ctrough_) ^a^  < 664 ng/mL (4xPAIC_90_) ^b^  ≤ 166 ng/mL (PAIC_90_) | 14 (56%)  6 (24%)  2 (8%) | 12 (60%)  4 (20%)  2 (10%) | 2 (40%)  2 (40%)  - |
| PWH with ≥ 2 measurements  < 1120 ng/mL (Q1_Ctrough_) ^a^  < 664 ng/mL (4xPAIC_90_) ^b^  ≤ 166 ng/mL (PAIC_90_) | 5 (20%)  3 (12%)  - | 5 (25%)  3 (15%)  - | -  -  - |
| Rilpivirine q8w | | | |
| Plasma concentrations  < 50 ng/mL (4xPAIC_90_) ^c^  < 32 ng/mL (Q1_Ctrough_) ^a^  ≤ 12 ng/mL (PAIC_90_) | 27 (35%)  12 (16%)  1 (1%) | 18 (30%)  8 (13%)  - | 9 (56%)  4 (25%)  1 (6%) |
| PWH with ≥ 1 measurement  < 50 ng/mL (4xPAIC_90_) ^c^  < 32 ng/mL (Q1_Ctrough_) ^a^  ≤ 12 ng/mL (PAIC_90_) | 16 (64%)  9 (36%)  1 (4%) | 11 (55%)  5 (25%)  - | 5 (100%)  4 (80%)  1 (20%) |
| PWH with ≥ 2 measurements  < 50 ng/mL (4xPAIC_90_) ^c^  < 32 ng/mL (Q1_Ctrough_) ^a^  ≤ 12 ng/mL (PAIC_90_) | 7 (28%)  2 (8%)  - | 5 (25%)  2 (10%)  - | 2 (40%)  -  - |

PWH: people with HIV; BMI: body-mass index; q8w: every 8 weeks.

^a^ Correspond to the limit of the first quartile (25^th^ percentile) when considering all rilpivirine and cabotegravir concentrations observed in phase III FLAIR^4^ and ATLAS.^13^

^b^ Corresponds to 4xPAIC_90_,^8^ and corresponds also to the 5^th^ percentile of cabotegravir long-acting initial Ctrough observed in PrEP prevention studies.^12^

^c^ Minimum concentration to ensure optimal therapeutic response.^9,10^

# Table S4: Results of the mixed-effect regression on trough concentrations of cabotegravir and rilpivirine.

| Cabotegravir | | | |
| --- | --- | --- | --- |
| Fixed effects | **Coefficients**  **[CI_95%_]** | **Geometric mean ratio per one unit increase [CI_95%_]** | **p-value** |
| Intercept (β_0_)  Male (β_1_)  BMI (β_2_)  Albumin plasma level (β_3_)  Week 8 (β_4_)  Week 16 (β_5_)  Week 24 (β_6_)  Week 32 (β_7_) | 7.40 [7.10 to 7.60]  -0.40 [-0.70 to -0.20]  0.00 [-0.02 to 0.02]  0.01 [-0.01 to 0.02]  0.40 [0.20 to 0.60]  -0.20 [-0.40 to 0.00]  -0.01 [-0.20 to 0.20]  -0.03 [-0.30 to 0.20] | 0.65 [0.53 – 0.80]  1.00 [0.98 – 1.00]  1.00 [0.99 – 1.00]  1.40 [1.20 – 1.80]  0.82 [0.68 – 1.00]  0.99 [0.81 – 1.20]  0.97 [0.77 – 1.20] | < 0.01  0.96  0.46  < 0.01  0.05  0.93  0.78 |
| Random effects | **Standard deviation**  **[CI_95%_]** | **Geometric coefficient of variation [CI_95%_]** | **p-value** |
| Individual (intercept)  Residual | 0.30 [0.20 to 0.40]  0.50 [0.50 to 0.60] | 0.36 [0.25 – 0.46]  0.68 [0.62 – 0.77] | - |

| Rilpivirine | | | |
| --- | --- | --- | --- |
| Fixed effects | **Coefficients**  **[CI_95%_]** | **Geometric mean ratio per one unit increase [CI_95%_]** | **p-value** |
| Intercept (β_0_)  Male (β_1_)  BMI (β_2_)  Albumin plasma level (β_3_)  Week 8 (β_4_)  Week 16 (β_5_)  Week 24 (β_6_)  Week 32 (β_7_) | 4.00 [3.80 to 4.20]  0.10 [-0.10 to 0.30]  0.01 [-0.01 to 0.03]  0.01 [-0.00 to 0.02]  -0.20 [-0.30 to -0.02]  -0.40 [-0.60 to -0.30]  -0.30 [-0.50 to -0.20]  -0.20 [-0.30 to -0.03] | 1.10 [0.90 – 1.30]  1.00 [0.99 – 1.00]  1.00 [1.00 – 1.00]  0.86 [0.76 – 0.98]  0.65 [0.57 – 0.74]  0.70 [0.63 – 0.82]  0.84 [0.73 – 0.97] | 0.42  0.37  0.17  0.03  < 0.01  < 0.01  0.02 |
| Random effects | **Standard deviation**  **[CI_95%_]** | **Geometric coefficient of variation [CI_95%_]** | **p-value** |
| Individual (intercept)  Residual | 0.40 [0.30 to 0.40]  0.30 [0.30 to 0.30] | 0.42 [0.35 – 0.49]  0.34 [0.31 – 0.38] | - |

CI_95%_: 95% confidence interval; β: parameter coefficient. BMI and albumin plasma levels were centered on their median, i.e., 25.5 kg/m^2^ and 46 g/L, respectively.

Note: The assumptions underlying linear mixed-effects model appeared reasonably supported by the data in these exploratory analyses.

# Table S5: Logistic regression between detectable HIV RNA and drug concentrations at trough after intramuscular administration.

| Cabotegravir | | | |
| --- | --- | --- | --- |
| Detectable HIV ≥ 20 copies/mL | **Coefficients**  **[CI_95%_]** | **Odds ratio**  **[CI_95%_]** | **p-value** |
| Intercept (β_0_)  Trough concentration (β_1_) | 0.50 [-2.40 to 3.20]  -0.30 [-0.80 to 0.10] | 0.70 [0.50 – 1.10] | 0.10 |
| Detectable HIV ≥ 50 copies/mL | **Coefficients**  **[CI_95%_]** | **Odds ratio**  **[CI_95%_]** | **p-value** |
| Intercept (β_0_)  Trough concentration (β_1_) | -6.80 [-15.20 to 0.20]  0.40 [-0.60 to 1.60] | 1.50 [0.60 – 4.80] | 0.44 |

| Rilpivirine | | | |
| --- | --- | --- | --- |
| Detectable HIV ≥ 20 copies/mL | **Coefficients**  **[CI_95%_]** | **Odds ratio**  **[CI_95%_]** | **p-value** |
| Intercept (β_0_)  Trough concentration (β_1_) | -0.70 [-2.80 to 1.40]  -0.30 [-0.90 to 0.20] | 0.70 [0.40 – 1.30] | 0.24 |
| Detectable HIV ≥ 50 copies/mL | **Coefficients**  **[CI_95%_]** | **Odds ratio**  **[CI_95%_]** | **p-value** |
| Intercept (β_0_)  Trough concentration (β_1_) | -1.60 [-6.50 to 2.40]  -0.60 [-1.70 to 0.70] | 0.60 [0.20 – 2.00] | 0.35 |

CI_95%_: 95% confidence interval; β: parameter coefficient.
